# Journal data-sharing policies and its impact in publications: A cross-sectional study protocol

**DOI:** 10.1371/journal.pone.0331697

**Published:** 2025-09-02

**Authors:** Pinge Zhao, Xin Zhang, Liandi Dai, Baoguo Ma, Yuting Duan, Yan Xu, Hongmei Wei, Shengwei Wu, Linghui Xiong

**Affiliations:** 1 The Affiliated Brain Hospital, Guangzhou Medical University, Key Laboratory of Neurogenetics and Channelopathies of Guangdong Province and the Ministry of Education of China, Guangzhou Medical University, Guangzhou, People's Republic of China; 2 Evidence-based Medicine Center, The Affiliated Traditional Chinese Medicine Hospital, Guangzhou Medical University, Guangzhou, People's Republic of China; 3 Clinical School of Integrated Traditional Chinese and Western Medicine, Guangzhou Medical University, Guangzhou, People's Republic of China; 4 The Affiliated Guangzhou Hospital of TCM of Guangzhou University of Chinese Medicine, Guangzhou, People's Republic of China; Dental Hypothesis, IRAN, ISLAMIC REPUBLIC OF

## Abstract

Responsible data sharing in clinical research can enhance the transparency and reproducibility of research evidence, thereby increasing the overall value of research. Since 2024, more than 5,000 journals have adhered to the International Committee of Medical Journal Editors (ICMJE) Data Sharing Statement (DSS) to promote data sharing. However, due to the significant effort required for data sharing and the scarcity of academic rewards, data availability in clinical research remains suboptimal. This study aims to explore the impact of biomedical journal policies and available supporting information on the implementation of data availability in clinical research publications This cross-sectional study will select 303 journals and their latest publications as samples from the biomedical journals listed in the Web of Science Journal Citation Reports based on stratified random sampling according to the 2023 Journal Impact Factor (JIF). Two researchers will independently extract journal data-sharing policies from the submission guidelines of eligible journals and data-sharing details from publications using a pre-designed form from Apr 2025 to Dec 2025. The data sharing levels of publications will be based on the openness of the data-sharing mechanism. Binomial logistic regression analyses will be used to identify potential journal factors that affect publication data-sharing levels. This protocol has been registered in Open Science Framework (OSF) Registries: https://doi.org/10.17605/OSF.IO/EX6DV.

## Introduction

Responsible sharing of data from clinical trials can increase the transparency of study evidence, provide opportunities for researchers to strengthen their study designs, validate study findings, and generate new key findings through the synthesis of data sharing, which maximizes scientific knowledge for researchers and participants in clinical trials [1]. However, data sharing in clinical research publications is unsatisfactory, with a prevalence of actual data sharing of only 2% between 2016 and 2021 [2]. The absence and opacity of large amounts of clinical trial data may be detrimental to developing more efficient and effective public health service [3]. Therefore, an increasing number of biomedical scientists and stakeholders advocated for data sharing policy [4–6].

However, promoting data sharing is challenging and fraught with technical, motivational, economic, political, legal, and ethical barriers [3]. Specifically, while a standard de-identification process can reduce the risk of patient information being re-identified [7], the lack of regulation of shared data use can lead to concerns about inappropriate reuse and re-identification [8,9]. Secondly, due to the chronic lack of resources in clinical practice (e.g., time, staff, funding), researchers prioritize publishing study results over sharing data [10], as academic publications rather than data ownership represent scientific productivity and capability for authors [11]. Finally, the author’s concerns about losing the advantages of secondary publication, unwarranted questions about the authenticity and integrity of shared data [12], and the lack of a simple, uniform, and efficient data-sharing process hinder data sharing in clinical research [13].

Current measures to promote data sharing include emphasizing data authorship [14], funding data-sharing platforms (e.g., data repositories, online sharing platforms) [15], and journal data-sharing requirements [16]. However, the contribution of data authors is insufficiently recognized, and data authorship is rarely rewarded with academic recognition and rewards [17,18]. Funding for data repositories does not directly incentivize researchers to share data. Under publication pressure, journal data-sharing requirements seem to be a coin step in promoting data sharing [19].

In 2017, the International Committee of Medical Journal Editors (ICMJE) required that publications should include a specific Data Sharing Statement (DSS) to promote new norms of data sharing [20]. More than 5,000 journals followed the ICMJE recommendations [16], but around 56% of journals did not state a specific data-sharing policy, possibly due to concerns about increased costs and loss of submissions [21]. However, studies have found that the strength of journal requirements of policies (e.g., encourage, mandatory) [22] and the availability of resources (e.g., free data repository links, data-sharing handbook) may influence publications adherence to journal policies [23]. To date, it lacks comprehensive surveys on the impact of journal policies on publication data-sharing implementations.

Hence, we undertake this research to extract journal policy data and available supporting information from submission guidelines and assess the data-sharing implementations from the journal’s publication to explore the potential impact of journal data-sharing policies on the publication data-sharing level. We hope this research will offer a deeper insight into the data-sharing policies and promote the further development of the data-sharing new normal.

## Methods

This is an analytical, cross-sectional, descriptive study. Data collection will be carried out in biomedical journals (from Web of Science Journal Citation Reports [24]) by two experienced researchers. We will gather detailed data-sharing policies and data-sharing statements from journal submission guidelines and its latest publications. This protocol is registered and publicly available via the Open Science Framework [25] (Registration DOI: https://doi.org/10.17605/OSF.IO/EX6DV). This study will follow STORBE guidelines for cross-sectional study [26].

### Sample size calculation

The minimum sample size (N) for this study was estimated by Events Per Variable (EPV) formula $N=\frac{k*EPV}{p}$ [27] and the formula $N=\frac{1.96^{2}p(1-p)}{E^{2}}$ (for binary outcome) [28]. This was conducted to ensure that sufficient power will be available to obtain journal adherence levels for data sharing. When hypothesize EPV = 10, our study has one independent variable and 8 co-variables (*K*), and the proportion (*p*) of journals that did not required data-sharing statements is 0.27 [16], we generate the sample size is 297. When we set 0.05 for a margin error (*E*), the proportion (*p*) is 0.27 [16], we get the hypotheses result in sample sizes is 303. Therefore, we will use the largest sample size of 303 in this study.

### Data source and sampling methods

Since the Web of Science Journal Citation Reports only supports exporting 600 records at a time, we exported journals individually according to their category (biomedical journals), then excluded duplicate journals (a journal may have multiple category labels) by International Standard Serial Number (ISSN)/ eISSN to finalize the identification of 6313 journals We sorted and coded the journals according to Journal Impact Factors (2023 JIF). Details of the coding are in S1 Appendix.

We will fulfill stratified sampling based on the 2023 JIF, and the block group lengths will be set to NA- < 1.0, 1.0- < 3.0, 3.0- < 5.0, 5.0- < 10.0, 10.0- < 20.0, ≥ 20.0. If the extracted journals do not meet the inclusion criteria, we will use the pre-specified replacement hierarchy. Specifically, the first principle is to use journals with the same JIF as the ineligible journals instead, and the second principle is to use the code closest to the ineligible journal when multiple journals with the same JIF exist (prioritizing the next code, or the previous code if the JIFs are different). Any details of the replacement will be published along with the publication.

### Eligibility criteria

Journal inclusion criteria: (1) biomedical journal; (2) published at least one clinical research in the latest issue in 2024; (3) English journals; (4) peer-reviewed journals. Journal exclusion criteria: (1) publish only conference papers; (2) without submission guidelines, (3) not publish clinical studies.

Studies will be the latest clinical research publications in the latest issue of eligible journals. The specific order of clinical research [29] and the Oxford Centre for Evidence-based Medicine levels [30] (see S2 Appendix), which will be: (1) randomized controlled trials, (2) non-randomized studies (quasi-experiment, field trial, community trial), (3) cohort studies, (4) case-control studies, (5) cross-sectional studies, (6) case reports, (7) case series, (8) surveillance studies, and (9) qualitative research. The exclusion criteria will be (1) full text unavailable; (2) non-human studies; (3) non-English publications.

Two researchers (XZ and BM) will independently check journal homepages and their latest issues to find eligible journals and studies. Then, we will record the journal submission guidelines link and download the study’s full text. Any discrepancies will be discussed and resolved with the senior researcher (PZ).

### Study status and timeline

Data will be collected from Apr 2025 through Dec 2025 (the detailed data extraction time for each journal will be presented in future studies). Two researchers (XZ and BM) will extract data independently in a standardized data extraction form in Microsoft Excel 2019 (Microsoft Excel 2019 MSO 2210 Build 16.0.15726.20188 32). Any discrepancy in the data extraction will be resolved via discussion or adjudication by the senior reviewer (PZ). Before the formal data collection, we will perform at least three rounds of pilot testing on at least 10 journals and 10 studies to ensure consistency and accuracy, and only the kappa coefficients > 0.90 will start the formal extraction.

### Data extraction

The following information of journals will be extracted: (1) journal name, (2) JCR abbreviation, (3) publisher, (4) ISSN, (5) eISSN, (6) category, (7) 2023 JIF, (8) JIF quartile, (9) percentage of citable Open Assess (OA), (10) whether the submission guidelines contain a data availability statements/ data-sharing statements, (11) follows what kind of data policy (e.g., Springer Nature research data policy), (12) requirements level of data-sharing (e.g., encourage, should, required, must), (13) any indication of what should be included in data sharing, (14) guidelines for sharing data, (15) are data repositories recommended, (16) guidelines/ links for data repositories, and (17) whether easy to obtain data sharing policies.

Data extraction in studies: (1) volume of journal, (2) publish date, (3) name, (4) first author, (5) research type, (6) registry, (7) funding, (8) interest, (9)whether it contains a data sharing statement. Then we will used the ICMJE DSS [20] to collect possible structures of data availability of the study, which contains: (1) will individual participant data be available, (2) what data in particular will be shared, (3) what other documents will be available, (4) when will data be available, (5) with whom, (6) for what types of analyses, (7) by what mechanism will data be available. Finally, we will grade the data sharing levels according to the openness of the data-sharing mechanism. We will define data-sharing with raw data provided in open-access repositories/ appendix as “high level of data-sharing”, data can be available by contacting authors with reasonable requests or data will be available at additional time (e.g., one year after publication), no mention of data-sharing mechanism as “small level of data-sharing”, and without data-sharing as “without data-sharing”.

### Data clean and confounder identification

We will classify the requirements level of data-sharing policies as “high” (mandatory data sharing), “small” (encouraging data sharing), and “no” (no mention of data sharing) to explore the potential impact of policies on data-sharing level. We identified all variables that may be involved in the causal effects of journal data sharing policies and data sharing practices by discussing with methodological experts with prior knowledge, and presented them in a directed acyclic graphs (DAGitty v3.1) [31] (see S3 Appendix). The following co-variables will be considered for inclusion in the final model: journal JIF, percentage of citable OA, whether easy to obtain data sharing policies, data sharing supporting information (requirements level of data-sharing, indication of data-sharing, guidelines for sharing data, data repositories recommendation, and guidelines/ links for data repositories).

### Data analysis

The analyses and stratified sampling will be conducted in R software (Version R 4.4.3; R Core Team, 2025; R Foundation for Statistical Computing, Vienna, Austria). The primary outcome is to explore the journal data-sharing policies, available supporting information, and data-sharing level in publications. Then we will further explore the potential impact of data-sharing policy on the data-sharing level. Statistical significance will be defended with two-sided 95% Confidence Intervals (CIs), P < 0.05.

Descriptive statistics will be presented in counts (n) and percentages (%) by charts. We will describe the journal data-sharing policies and supporting information at the journal level. Describe the specific data-sharing details at the publication level.

Binomial logistic regression analyses will be used to estimate the adjusted odds ratios (aORs) and 95% confidence intervals (CIs) of the data-sharing policy with data-sharing level. We will use data-sharing policy as the dependent variable, use data-sharing level as the independent variable, and journal JIF, percentage of citable OA, whether easy to obtain data sharing policies, data sharing supporting information (requirements level of data-sharing, indication of data-sharing, guidelines for sharing data, data repositories recommendation, and guidelines/ links for data repositories) as the co-variables.

## Discussion

Good data sharing and management should meet Findability, Accessibility, Interoperability, and Reusability (FAIR) principles to maximize the data value from clinical trials [32]. However, responsible data sharing requires significant efforts in data collection, archival, sharing, and long-term maintenance, but the incentives for data sharing are scarce [14]. Those disproportionate data-sharing efforts and incentives significantly affect data sharing in clinical research and may persist in modern scientific evaluation systems [17]. Therefore, it is critical to provide extra incentives and assistance for authors to promote data sharing. Our study will explore potential factors at the journal level.

It is known that journal data-sharing policies are requirements rather than incentives for authors. Current journal data-sharing policies typically require a declaration of data-sharing/ data availability statements without any defining details about data accessibility [33]. Under publication pressure, 52%−96% of biological publications contained a data-sharing statement, but 76.6% − 93% of studies did not share data after published studies [34,35]. Declarations of various reasons for data unavailability in the DSS, not responding or declining to the requests for the data, and responding to data assessment requests but failing to provide data are the most common types of data unavailability [34–36]. Therefore, developing more detailed data-sharing policies and providing additional support for data-sharing, such as guidelines for the use of open data repositories, professional de-identification process training, and technical support for data-sharing, may be able to facilitate data-sharing.

### Strengths and limitations of the study

The strengths of this study are: (1) There will be no limitations of biomedical journal categories and disease types of the included samples, allowing for a more comprehensive reflection of the data sharing policies and actual data-sharing practices in clinical research. (2) We systematically explored details related to the strength of data sharing requirements and accompanying supporting information for journals, which may be beneficial to the development of journal data sharing policies. (3) This will be the first study to explore the journal data-sharing policies and actual data-sharing practices in their clinical research publications, thereby establishing a solid foundation for data sharing as the new normal in clinical research in the future.

The limitations of this study are: (1) The lag in clinical research publications. While we will include the most recent publications from journals, some journals may have received their most recent publications in a period before the policy update (especially for journals with a high volume of submissions but fewer publications). Those publications may not provide timely feedback on the journal’s policy requirements, depending on whether or not the journal will review incoming manuscripts again for formatting before publication. (2) We will only include journals with official websites in English; study findings may not be generalizable to journals published in other languages. (3) We may be unable to avoid subjective bias in policy interpretation, even if we standardize inter-assessor agreement between reviewers. Analysis of study outcomes should be interpreted with caution.

## Conclusion

This study serves as the inaugural examination of the application of journal data-sharing policies and available supporting information to publications. The insights drawn from this study may have immense potential to promote the further development of the data-sharing new normal.

## Supporting information

S1 Appendix Journals code. (XLSX)

S2 Appendix The specific order of included studies. (DOCX)

S3 Appendix DAGs. (DOCX)

## References

[1] Institute of Medicine. Sharing clinical trial data: maximizing benefits, minimizing risk. The National Academies Press; 2015. 25590113

[2] Hamilton DG, Hong K, Fraser H, Rowhani-Farid A, Fidler F, Page MJ. Prevalence and predictors of data and code sharing in the medical and health sciences: systematic review with meta-analysis of individual participant data. BMJ. 2023;382:e075767. doi: 10.1136/bmj-2023-075767 37433624 PMC10334349

[3] van Panhuis WG, Paul P, Emerson C, Grefenstette J, Wilder R, Herbst AJ, et al. A systematic review of barriers to data sharing in public health. BMC Public Health. 2014;14:1144. doi: 10.1186/1471-2458-14-1144 25377061 PMC4239377

[4] DeVito NJ, French L, Goldacre B. Noncommercial funders’ policies on trial registration, access to summary results, and individual patient data availability. JAMA. 2018;319(16):1721–3. doi: 10.1001/jama.2018.2841 29710154 PMC5933390

[5] Kiley R, Peatfield T, Hansen J, Reddington F. Data sharing from clinical trials - a research funder’s perspective. N Engl J Med. 2017;377(20):1990–2. doi: 10.1056/NEJMsb1708278 29141170

[6] Hamilton DG, Fraser H, Hoekstra R, Fidler F. Journal policies and editors’ opinions on peer review. Elife. 2020;9:e62529. doi: 10.7554/eLife.62529 33211009 PMC7717900

[7] Rights (OCR) O for C. Guidance Regarding Methods for De-identification of Protected Health Information in Accordance with the Health Insurance Portability and Accountability Act (HIPAA) Privacy Rule. [cited 10 Dec 2024]. Available from: https://www.hhs.gov/hipaa/for-professionals/privacy/special-topics/de-identification/index.html

[8] McGraw D. Building public trust in uses of health insurance portability and accountability act de-identified data. J Am Med Inform Assoc. 2013;20(1):29–34. doi: 10.1136/amiajnl-2012-000936 22735615 PMC3555317

[9] Watson H, Gallifant J, Lai Y, Radunsky AP, Villanueva C, Martinez N, et al. Delivering on NIH data sharing requirements: avoiding Open Data in Appearance Only. BMJ Health Care Inform. 2023;30(1):e100771. doi: 10.1136/bmjhci-2023-100771 37344002 PMC10314418

[10] Danchev V, Min Y, Borghi J, Baiocchi M, Ioannidis JPA. Evaluation of data sharing after implementation of the International Committee of Medical Journal Editors Data Sharing Statement Requirement. JAMA Netw Open. 2021;4(1):e2033972. doi: 10.1001/jamanetworkopen.2020.33972 33507256 PMC7844597

[11] Neill US. Publish or perish, but at what cost? J Clin Invest. 2008;118(7):2368. doi: 10.1172/JCI36371 18596904 PMC2439458

[12] Rathi V, Dzara K, Gross CP, Hrynaszkiewicz I, Joffe S, Krumholz HM, et al. Sharing of clinical trial data among trialists: a cross sectional survey. BMJ. 2012;345(nov20 3):e7570. doi: 10.1136/bmj.e7570 PMC3502744 23169870

[13] Anderson JM, Johnson A, Rauh S, Johnson B, Bouvette M, Pinero I, et al. Perceptions and opinions towards data-sharing: a survey of addiction journal editorial board members. J Sci Pract Integr. 2022;2022. doi: 10.35122/001c.35597 38804666 PMC11129878

[14] Bierer BE, Crosas M, Pierce HH. Data authorship as an incentive to data sharing. N Engl J Med. 2017;376(17):1684–7. doi: 10.1056/NEJMsb1616595 28402238

[15] Dey P, Ross JS, Ritchie JD, Desai NR, Bhavnani SP, Krumholz HM. Data sharing and cardiology: platforms and possibilities. J Am Coll Cardiol. 2017;70(24):3018–25. doi: 10.1016/j.jacc.2017.10.037 29241491

[16] Zhang J, Liu Y, Thabane L, Li J, Bai X, Li L, et al. Journal requirement for data sharing statements in clinical trials: a cross-sectional study. J Clin Epidemiol. 2024;172:111405. doi: 10.1016/j.jclinepi.2024.111405 38838963

[17] Time to recognize authorship of open data. Nature. 2022;604(7904):8. doi: 10.1038/d41586-022-00921-x 35388202

[18] Devriendt T, Borry P, Shabani M. Credit and recognition for contributions to data-sharing platforms among cohort holders and platform developers in europe: interview study. J Med Internet Res. 2022;24(1):e25983. doi: 10.2196/25983 35023849 PMC8796038

[19] Ohmann C, Banzi R, Canham S, Battaglia S, Matei M, Ariyo C, et al. Sharing and reuse of individual participant data from clinical trials: principles and recommendations. BMJ Open. 2017;7(12):e018647. doi: 10.1136/bmjopen-2017-018647 29247106 PMC5736032

[20] Taichman DB, Sahni P, Pinborg A, Peiperl L, Laine C, James A, et al. Data Sharing Statements for Clinical Trials - A Requirement of the International Committee of Medical Journal Editors. N Engl J Med. 2017;376(23):2277–9. doi: 10.1056/NEJMe1705439 28581902

[21] Siebert M, Gaba JF, Caquelin L, Gouraud H, Dupuy A, Moher D, et al. Data-sharing recommendations in biomedical journals and randomised controlled trials: an audit of journals following the ICMJE recommendations. BMJ Open. 2020;10(5):e038887. doi: 10.1136/bmjopen-2020-038887 32474433 PMC7264700

[22] Agha RA, Fowler AJ, Limb C, Whitehurst K, Coe R, Sagoo H, et al. Impact of the mandatory implementation of reporting guidelines on reporting quality in a surgical journal: a before and after study. Int J Surg. 2016;30:169–72. doi: 10.1016/j.ijsu.2016.04.032 27112835

[23] Hepkema WM, Horbach SPJM, Hoek JM, Halffman W. Misidentified biomedical resources: journal guidelines are not a quick fix. Intl J Cancer. 2021;150(8):1233–43. doi: 10.1002/ijc.33882 PMC9300184 34807460

[24] Web of Science—Journal Citation Reports. [cited 10 Dec 2024]. https://www.webofscience.com/

[25] OSF. [cited 10 Dec 2024]. 10.17605/OSF.IO/EX6DV

[26] STROBE Statement—Checklist of items that should be included in reports of cross-sectional studies. [cited 10 Dec 2024]. http://www.plosmedicine.org/10.1007/s00038-007-0239-9 18522360

[27] Peduzzi P, Concato J, Kemper E, et al. A simulation study of the number of events per variable in logistic regression analysis. J Clin Epidemiol. 2023. 10.1016/s0895-4356(96)00236-3 8970487

[28] Riley RD, Ensor J, Snell KIE, Harrell FE Jr, Martin GP, Reitsma JB, et al. Calculating the sample size required for developing a clinical prediction model. BMJ. 2020;368:m441. doi: 10.1136/bmj.m441 32188600

[29] Kiani AK, Naureen Z, Pheby D, Henehan G, Brown R, Sieving P, et al. Methodology for clinical research. J Prev Med Hyg. 2022;63(2 Suppl 3):E267–78. doi: 10.15167/2421-4248/jpmh2022.63.2S3.2769 36479476 PMC9710407

[30] Oxford Centre for Evidence based Medicine. Oxford Centre for Evidence-Based Medicine: Levels of Evidence. [cited 10 Dec 2024]. https://www.cebm.ox.ac.uk/resources/levels-of-evidence/oxford-centre-for-evidence-based-medicine-levels-of-evidence-march-2009

[31] DAGitty v3.1 [cited 10 Dec 2024]. https://dagitty.net/dags.html#

[32] Wilkinson MD, Dumontier M, Jan Aalbersberg I, Appleton G, Axton M, Baak A, et al. Addendum: The FAIR Guiding Principles for scientific data management and stewardship. Sci Data. 2019;6(1):6. doi: 10.1038/s41597-019-0009-6 30890711 PMC6427092

[33] Johnson AL, Anderson JM, Bouvette M, Pinero I, Rauh S, Johnson B, et al. Clinical trial data-sharing policies among journals, funding agencies, foundations, and other professional organizations: a scoping review. J Clin Epidemiol. 2023;154:42–55. doi: 10.1016/j.jclinepi.2022.11.009 36375641

[34] Asmundo MG, Durukan E, Russo GI, Jensen CFS, Østergren PB, Cimino S, et al. Data availability statements and data sharing in urology: a false promise? Eur Urol Focus. 2024;10(6):999–1002. doi: 10.1016/j.euf.2024.05.019 38839506

[35] Gabelica M, Bojčić R, Puljak L. Many researchers were not compliant with their published data sharing statement: a mixed-methods study. J Clin Epidemiol. 2022;150:33–41. doi: 10.1016/j.jclinepi.2022.05.019 35654271

[36] Gabelica M, Cavar J, Puljak L. Authors of trials from high-ranking anesthesiology journals were not willing to share raw data. J Clin Epidemiol. 2019;109:111–6. doi: 10.1016/j.jclinepi.2019.01.012 30738169

