# Supplementary material for: Journal data-sharing policies and its impact in publications: A cross-sectional study protocol
Source: PLoS One. 2025 Sep 2;20(9):e0331697. doi: 10.1371/journal.pone.0331697 (PMC12404463; doi:10.1371/journal.pone.0331697)

**Appendix 2 The specific order of included studies**

The specific order of included publications in this study will be: (1) randomized controlled trials, (2) non-randomized studies (quasi-experiment, field trial, community trial), (3) cohort studies, (4) case-control studies, (5) cross-sectional studies, (6) case reports, (7) case series, (8) surveillance studies, and (9) qualitative research.

Clinical trial: Randomized Clinical Trials, Parallel-Design Double-blind Trials, Crossover Trials, Equivalence and Noninferiority Trials, Cluster Trials, and Nonrandomized Clinical Trials


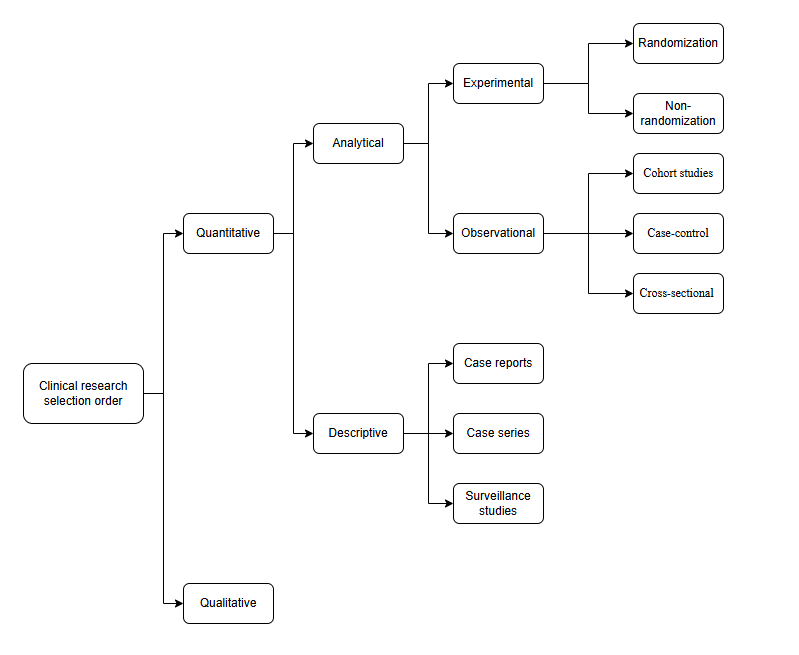

Supplement: S2 Appendix — (DOCX) [file pone.0331697.s002.docx]
