# Supplementary material for: Journal data-sharing policies and its impact in publications: A cross-sectional study protocol
Source: PLoS One. 2025 Sep 2;20(9):e0331697. doi: 10.1371/journal.pone.0331697 (PMC12404463; doi:10.1371/journal.pone.0331697)

**Appendix 3 Directed Acyclic Graphs**

We identify all variables involved in the causal effect of journal data sharing policies and data sharing practices by using prior knowledge and discussing with methodologists, and construct the ‘directed acyclic graphs’. The following co-variables will be considered for inclusion in the final model: journal JIF, percentage of citable OA, whether easy to obtain data sharing policies, data sharing supporting information (data sharing requirements level of data-sharing, indication of data-sharing, guidelines for sharing data, data repositories recommendation, and guidelines/ links for data repositories).


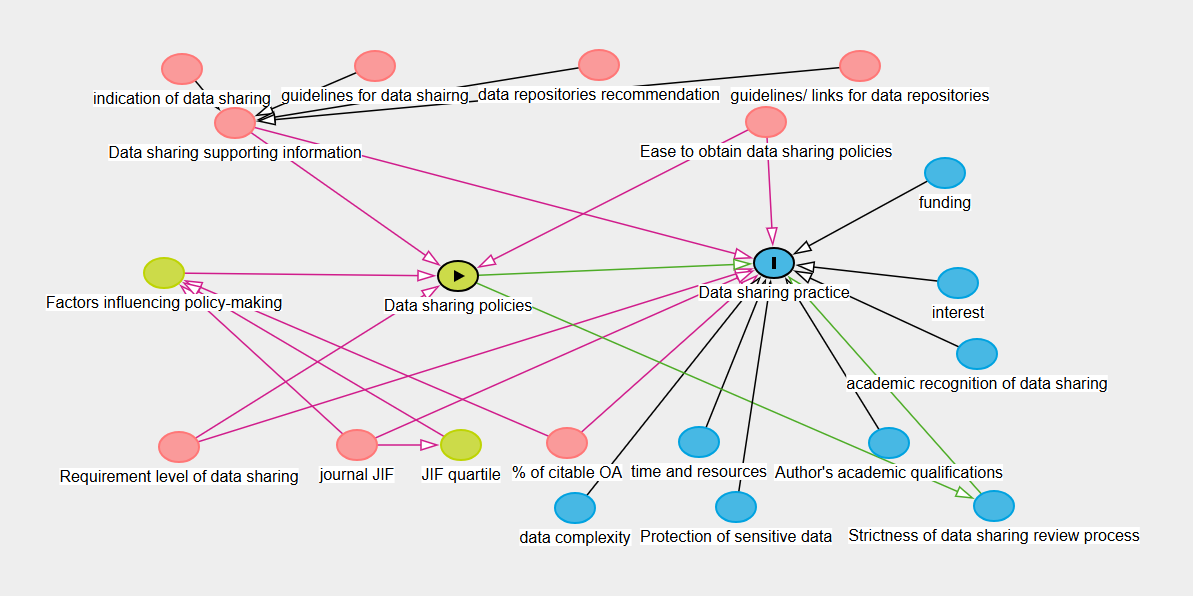


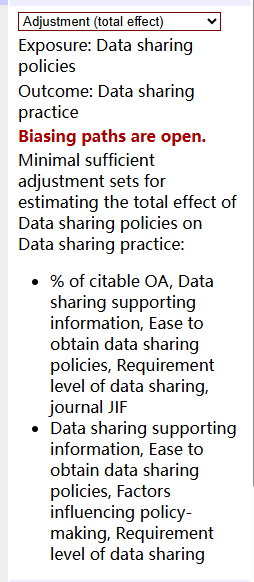

Supplement: S3 Appendix — (DOCX) [file pone.0331697.s003.docx]
